# Local care and treatment of liver disease (LOCATE) – A cluster-randomized feasibility study to discover, assess and manage early liver disease in primary care

**DOI:** 10.1371/journal.pone.0208798

**Published:** 2018-12-21

**Authors:** Magdy El-Gohary, Mike Moore, Paul Roderick, Emily Watkins, Joanne Dash, Tina Reinson, Colin Newell, Miranda Kim, Beth Stuart, Taeko Becque, Nick Sheron

**Affiliations:** 1 Primary Care and Population Sciences, School of Medicine, University of Southampton, Southampton, Hampshire, United Kingdom; 2 National Institute for Health Research, Southampton Biomedical Research Centre, University Hospital Southampton, Southampton, Hampshire, United Kingdom; Centre de Recherche en Cancerologie de Lyon, FRANCE

## Abstract

**Background:**

Chronic liver disease is an escalating problem both in the United Kingdom and worldwide. In the UK mortality rates have risen sharply over the previous 50 years predominantly due to alcohol, however the increasing prevalence of non-alcohol related fatty liver disease both in the UK and elsewhere is also of concern. Liver disease develops silently hence early detection of fibrosis is essential to prevent progression. Primary care presents an opportunity to identify at risk populations, however assessment largely comprises of indirect markers of fibrosis which have little prognostic value. We hypothesised that setting up nurse-led primary care based liver clinics using additional non-invasive testing would increase the number of new diagnoses of liver disease compared to usual care.

**Methods:**

This was a prospective, cluster randomised feasibility trial based in urban primary care in Southampton, United Kingdom. 10 GP practices were randomised to either intervention (liver health nurse) or control (care as usual). Pre recruitment audits were carried out in each practice to ascertain baseline prevalence of liver disease. Participants were subsequently recruited in intervention practices from July 2014-March 2016 via one of 3 pathways: GP referral, nurse led case finding based on risk factors or random AUDIT questionnaire mailouts. Liver assessment included the Southampton Traffic Light test (serum fibrosis markers HA and P3NP) and transient elastography (FibroScan). Cases were ascribed as ‘no fibrosis’, ‘liver warning’, ‘progressive fibrosis’ or ‘probable cirrhosis’. Post recruitment audits were repeated and incident liver diagnoses captured from July 2014-September 2016. Each new diagnosis was reviewed in a virtual clinic by a consultant hepatologist.

**Findings:**

910 participants were seen in the nurse led clinic—44 (4.8%) probable cirrhosis, 141 (15.5%) progressive fibrosis, 220 (24.2%) liver warning and 505 (55.5%) no evidence of liver fibrosis. 450 (49.5%) cases were due to NAFLD with 356 (39.1%) from alcohol. In the 405 with a liver disease diagnosis, 136 (33.6%) were referred by GP, 218 (53.8%) from nurse led case finding and 51 (12.6%) from the AUDIT mailout. 544 incident cases were identified in the intervention arm compared to 221 in the control arm in the period July 2014-September 2016 (adjusted odds ratio 2.4, 95% CI 2.1 to 2.8).

**Conclusions:**

The incorporation of a liver health nurse into GP practices was simple to arrange and yielded a much higher number of new diagnoses of liver disease compared to usual care. Nearly half of all participants recruited had a degree of liver disease. Nurse led case finding and GP referrals were most effective compared to AUDIT questionnaire mailouts in an urban population in identifying unknown disease. Utilising study and previous data allowed quick and effective virtual review by a hepatologist. Identifying those who are at risk of liver disease from harmful alcohol use remains a challenge and needs to be addressed in future work.

## Introduction

In 2014 the Lancet Commission on Liver Disease in the UK held as its primary recommendation: ‘Strengthen detection of early liver disease and its treatment by improving the level of expertise and facilities in primary care [1].’ Standardised mortality rates from liver disease have increased fourfold in the past few decades [2], predominantly due to alcoholic liver disease (ALD) and prompting the need for improvement in early detection in community settings as well as optimisation of treatment. The incidence of Non-Alcohol Related Fatty Liver Disease (NAFLD) is also rising as a result of increasing obesity [3]. Despite increasing recognition of the burden of liver disease, recent data from Public Health England (PHE) shows that hospital admission rates due to liver disease overall continues to increase year on year, as does admission from ALD specifically [4].

Liver disease progresses silently, with few if any signs or symptoms developing until cirrhosis and/or catastrophic complications of progressive liver fibrosis occur [5–7]. The early identification of developing liver disease is therefore paramount. In the case of ALD, which is the major cause of chronic liver disease (CLD) in the UK, total alcohol abstinence is the mainstay of treatment [8], and has shown to reduce complications and improve mortality even in advanced states [9–12]. However, a large number of patients–up to a third still die within a year of admission to hospital from alcohol related liver disease [12, 13]. Management of liver-related hospital admissions is also expensive; a recent economic evaluation in Scotland found that the excess lifetime cost of a patient with ALD is between £42,000 and £124,000 dependent upon levels of deprivation [14]. Identification and appropriate support at earlier stages therefore has the potential to improve mortality and reduce NHS costs.

A key difficulty lies in the accurate diagnosis of progressive liver fibrosis. The gold-standard test for many years was liver biopsy, expensive, invasive and with substantial risks [15]. Moreover, it is impractical to screen all those suspected of developing liver disease via biopsy, particularly those in community settings. However, primary care, which is ideally positioned to identify patients at risk of CLD, still largely relies on standard liver function tests (LFTs); namely alanine transaminase (ALT) and also ultrasonography, both of which are shown to have little prognostic value in identifying those at an earlier stage of fibrosis who go on to develop progressive disease and cirrhosis [16–18].

We previously developed the Southampton Traffic Light test (STL), an inexpensive test combining two established direct serum fibrosis markers–hyaluronic acid (HA) and amino terminal type III procollagen peptide (P3NP), together with platelet count to categorise a subject’s risk of liver disease [16]. A community study of the impact of diagnosing liver fibrosis using the STL showed half of the hazardous drinkers and two thirds of harmful drinkers with evidence of liver fibrosis had reduced their drinking by one AUDIT risk stage (harmful to hazardous, or hazardous to safe) at 1 year follow-up [19], suggesting such feedback may influence drinking behaviour. However this study based on postal screening of alcohol consumption from an unselected population had modest response rates and a high attrition rate in those responding.

Transient elastography (TE), (FibroScan; EchoSens) is a further non-invasive means of assessment of liver fibrosis [20]. This technique has shown to be highly sensitive in distinguishing between absent/mild fibrosis and severe fibrosis [18]. Although TE is largely used in hospital settings, there are now studies examining the acceptability and diagnostic accuracy of TE in primary care [21, 22] in identifying fibrotic liver disease.

We hypothesised that a relatively inexpensive nurse-led primary care based clinic could assess subjects at increased risk of liver disease and using these non-invasive tests to stage fibrosis / cirrhosis in each subject, substantially increase the number of new diagnoses of liver disease and determining the prevalence of liver fibrosis / cirrhosis in different risk groups. Furthermore by linking nurse led assessment to a virtual review of digital data by a consultant hepatologist, we felt it would be possible ascribe a liver disease aetiology and give each subject a treatment and follow up pathway, with minimum resource use compared to a routine hepatology outpatient appointment.

This paper presents a feasibility study of a cluster randomised trial to evaluate whether incorporating liver health nurses in GP practices improves the identification of progressive liver disease compared to usual care, by determining rates of recorded liver disease pre and post study period within intervention and control GP practices. It tests the feasibility of practice recruitment, patient identification by different routes, uptake of nurse assessment and non-invasive liver testing and yield and pattern of liver disease by severity and cause.

## Methods

This study was designed as a prospective cluster randomised feasibility trial in primary care, with five intervention and five control GP practices in Southampton, United Kingdom. Allocation of GP practices to either an intervention or control cluster was carried out via simple randomisation at a 1:1 ratio without matching. The ten practices were amongst those with the highest rates of liver admissions for any reason over the preceding few years to University Hospitals Southampton (UHS). Both groups undertook two audits of liver disease Read codes, one before the intervention and one after. Ethical approval was obtained from the Hampshire Research Authority, South Central—Hampshire A Research Ethics Committee.

Before recruitment commenced, an audit was carried out in all 10 practices by a liver health nurse employed by the practice to establish the prevalence of previously identified and possible significant liver disease. A Read code search strategy was developed in assistance with Explore Health Limited (Leeds, UK). Queries were run extracting patient level data from respective electronic patient records (EMIS Web, TPP SystmOne and Vision). Data pertaining to abnormal liver blood tests, risk factors for liver disease (evidence of hazardous/harmful alcohol intake and type 2 diabetes) and liver diagnoses were obtained for patients from 01/07/2007 to 30/06/2014 (prior to start of recruitment). Full audit queries are available in S1 Appendix.

**Fig 1:**
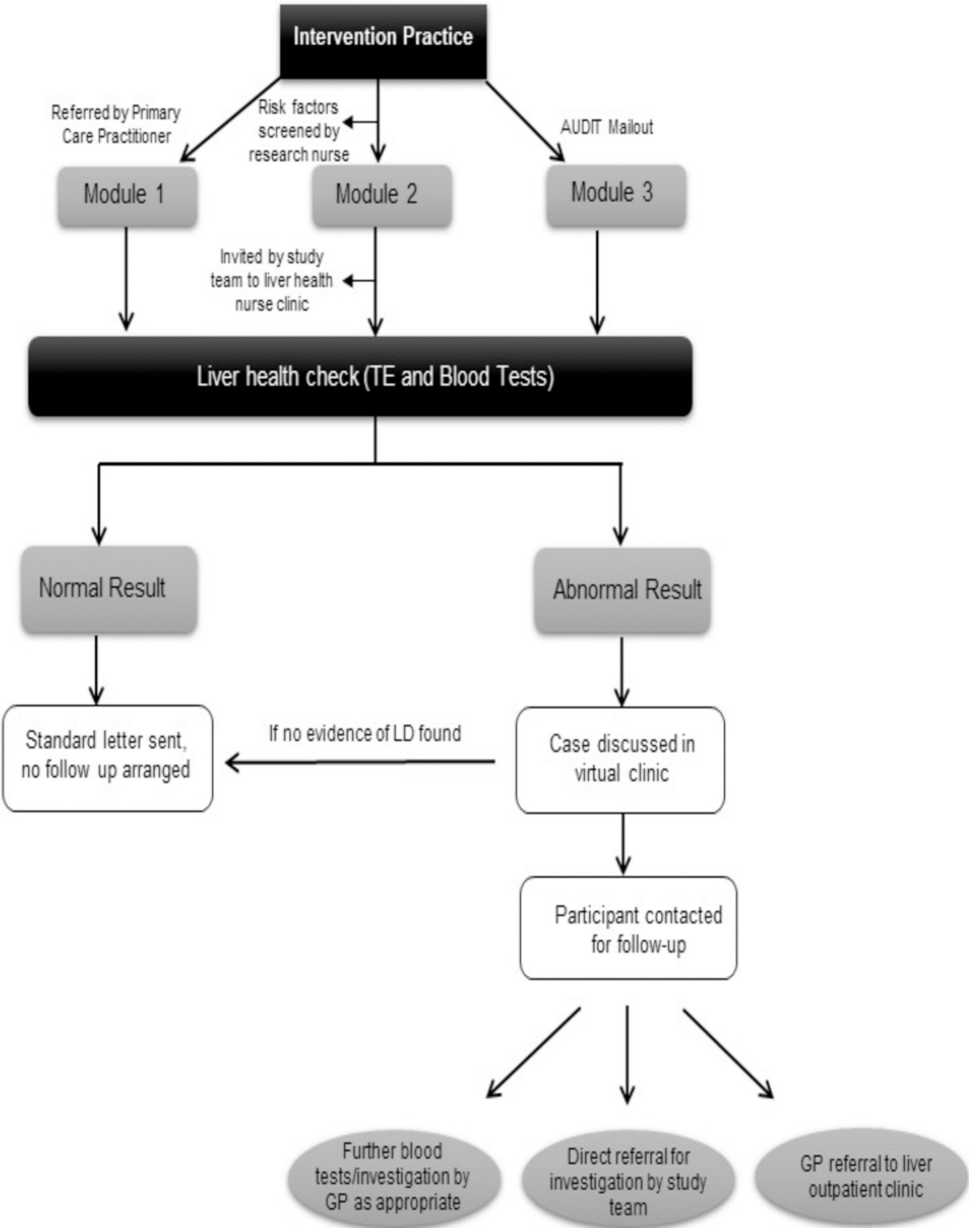
Identification of intervention practice participants and possible outcome.

**Fig 2:**
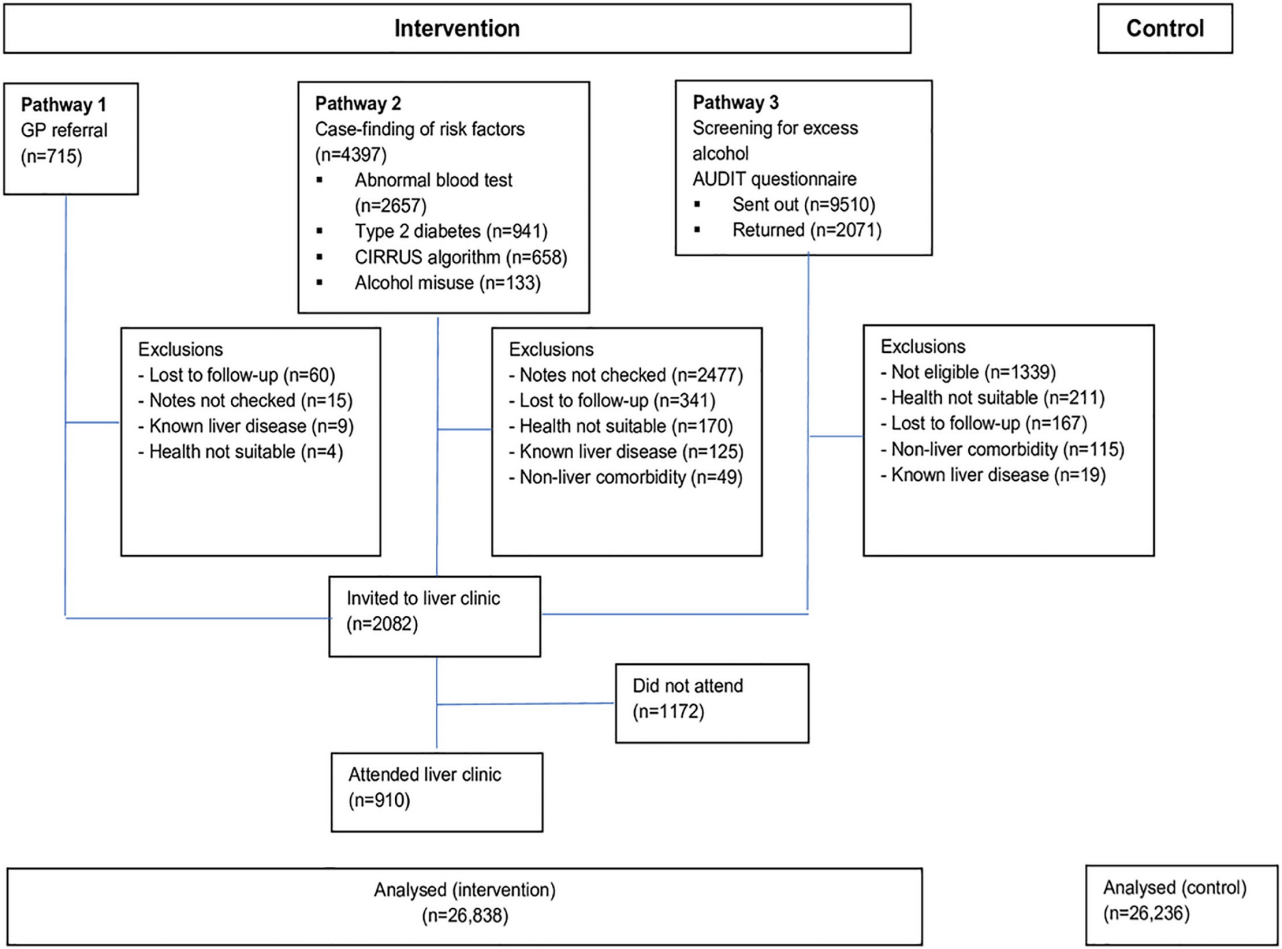
Flow of participants through the study.

The liver health nurse then recruited subjects from the intervention practices in the following risk categories (Figs 1 and 2): Subjects were recruited from July 2014 until March 2016.

Pathway 1: suspected cases opportunistically identified by GPs and practice nurses. Subjects were referred by practice staff and invitation to participate was then made by the liver health nurse. Participants who agreed to take part were subsequently seen in nurse led clinics.

Pathway 2: nurse led case finding of subjects with specific risk factors–elevated liver function tests, alcohol misuse or type 2 diabetes, using data in electronic clinical records (S1 Appendix). There are large numbers of subjects with type 2 diabetes or an isolated mild elevation of ALT, hence to limit numbers invited within the timescale of the study a random selection of subjects were selected for recruitment, for those with more severe or combined liver blood test abnormalities all subjects were considered for recruitment and were prioritised for invitation. The remainder of identified participants were seen on a ‘first come first serve’ basis. As part of the study we performed a pilot of a novel diagnostic algorithm (CIRRUS) to recruit subjects. Prior to invitation, notes that were screened by the liver health nurses for exclusion criteria were subsequently reviewed by the patient’s GP.

Pathway 3: population screening for excess alcohol use using AUDIT questionnaire [23] Invitation letters were sent to a random selection of subjects with an AUDIT questionnaire enclosed, similar to our previous study [19]. If the subject returned the questionnaire and scored 8 or higher they were invited into the study after screening for eligibility. Participants were free to distribute questionnaires to friends and family.

### Inclusion criteria

Minimum 18 years of age.

Registered patient with an intervention practice.

### Exclusion criteria

Significant enduring mental illness impeding informed consent to the study

Known terminal illness

Significant co-existing illness rendering participation difficult, e.g. housebound subjects or subjects undergoing cancer treatment

Pre-existing liver disease documented in primary care records

Participants were then invited to a clinic run by the liver health nurses at their GP surgery (Fig 2). All participants provided informed written consent. Blood pressure, BMI and waist circumference were measured. Blood samples for FBC, LFTs including AST and GGT were obtained in addition to the serum fibrosis markers HA and P3NP. All blood tests were analysed at the same NHS facility (UHS). In addition to these physiological markers of liver fibrosis, a physical parameter in liver stiffness was measured using TE performed with a portable FibroScan402 device (Echosens, France). A standard for a very reliable, or reliable liver stiffness evaluation was followed according to criteria suggested by Boursier *et al* [24]. Two different means of assessment were used as this approach has shown to increase negative predictive value in excluding significant fibrosis [25] as well as reduce the need for liver biopsy [26]. TE was pragmatically graded as follows: Median value of <6kPa = no fibrosis, 6-8kPa = ‘liver warning’, 8–12.9 kPa = ‘progressive fibrosis’, ≥13kPa = ‘probable cirrhosis’; the 13kPa cut off being relatively conservative and patients did not undergo a liver biopsy, hence the term probable.

**Fig 3:**
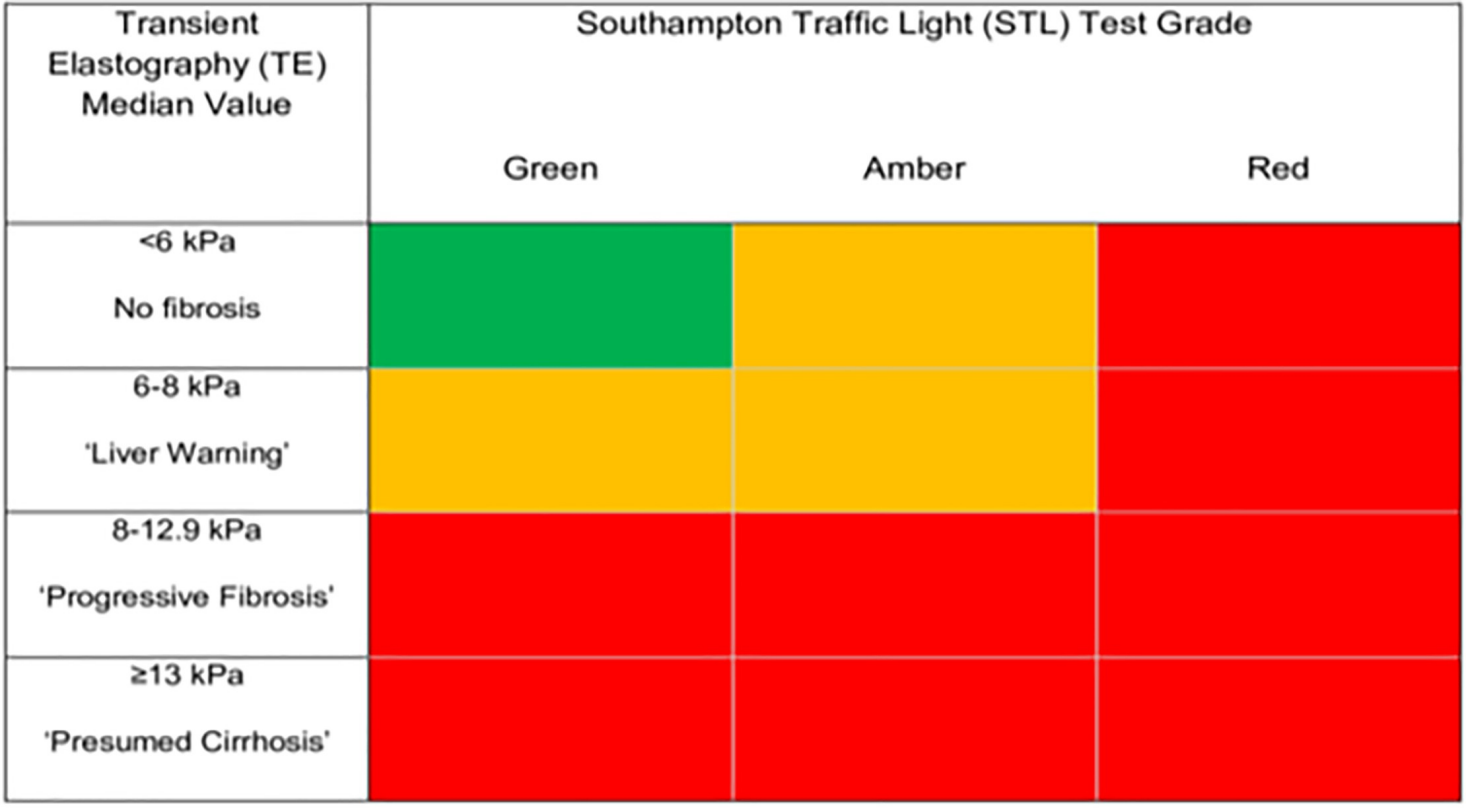
Green = no fibrosis, amber = ‘liver warning’, red = progressive fibrosis or cirrhosis.

The results of both tests were then combined and the maximum grade apportioned (Fig 3). In the event participants only had one modality assessed, the result of this was their final outcome.

The STL test (HA, P3NP, Platelet Count) used in the study was developed in Southampton alongside the commercial ELF test [27]. ELF utilises HA and P3NP, but with Tissue Inhibitor of Metalloproteinase (TIMP) in place of platelet count. In preliminary work for the study we compared STL and ELF test results in 597 patients: Spearman’s rank correlation coefficient was 0.90, and 149/150 positive ELF tests (ELF > 10.5) had a red STL result.

### Validation of diagnosis

Once the stage of fibrosis and related information was ascertained in the nurse led clinic, all subjects with evidence of liver fibrosis (probable cirrhosis, progressive fibrosis, liver warning), regardless of pathway into the study were assessed in a virtual combined clinic by a GP and consultant hepatologist. All relevant UHS tests, including study clinical data, fibrosis data and liver aetiology blood tests, were examined. Blood test data were visualised as multiple one graph / test plots, enabling the data trends for all the tests on the system to be analysed within a few minutes. Typically, 20 subjects were analysed in a session and a disease aetiology ascribed in most cases.

Where required, further additional tests were suggested to the GP. For example, in subjects diagnosed with cirrhosis, GPs were asked to refer the patient to UHS for an upper GI endoscopy. Where the diagnosis was uncertain or where secondary care treatment was required it was suggested to GPs that they refer the subject to UHS hepatology for further care or assessment. Where further tests were required a second or third virtual review was performed. Finally each subject was categorised in a simple matrix of aetiology vs stage of fibrosis / cirrhosis–the two key metrics needed to determine further management. GPs were informed of the final aetiology and disease severity and a management plan provided. Participants with no evidence of liver fibrosis were sent a letter outlining their findings, thanking them for taking part in the study and a copy of results sent to their GP.

Control practices continued to diagnose and manage liver disease as usual.

### Main outcome of feasibility study

Post study practice audits of liver disease were carried out following the final recruitment of participants in the intervention practices, and also within the control practices. This period captured incident cases of liver disease from 01/07/2014 to 30/09/2016 in each practice. Recruitment stopped in March 2016, with a 6 month period post recruitment included to allow coding to take place for incident cases.

### Statistical analysis

As this was a feasibility study, standard descriptive statistics were used to summarise variables: mean (SD) for continuous variables or median (IQR) for skewed variables, and numbers and percentages for categorical variables. For the comparison of liver disease identified in intervention and control practices, logistic regression analysis adjusting for baseline liver disease and clustering by GP practice was carried out. For the baseline and final analysis, only participants aged over 25 were included due to one practice with a large undergraduate student population. No formal statistical testing was undertaken, but differences between the two groups are presented with a 95% confidence interval. A sensitivity analysis utilising more advanced cases of liver disease was carried out to assess for differences between the control and intervention practices.

29 interviews with staff for qualitative analysis were also performed and will be reported separately.

## Results

Baseline characteristics for patients aged over 25 in each of the ten GP practices are presented in Table 1. There was some imbalance across randomised groups. The intervention practices had a higher proportion of males (55.5%) and slightly lower median age (44) than control practices (50.8% and 50 years). This was largely due to one intervention practice having a high population of university students. The rate of diabetes was lower in intervention practices (3.0% vs. 5.8%), and alcohol misuse slightly higher in intervention practices (4.2% vs. 3.0%). The prevalence of diagnosed liver disease prior to the start of the intervention was similar in both intervention and control practices at 1.3%. One control practice was taken over by new management during the study, was not able to provide follow up data, and was excluded from analysis.

**Table 1:** Baseline characteristics for patients aged over 25 with data at the start and end of trial.

Practice	Intervention						Control					
	A	B	C	D	E	INT	F	G	H	I	J	CON
Number	3,280	8,172	4,885	5,220	5,281	26,838	6,162	6,245	5,649	8,049	131	26,236
Gender–male, n	2,378	4,905	2,403	2,595	2,602	14,883	3,130	3,129	3,053	3,944	67	13,323
%	72.5	60.0	49.2	49.7	49.3	55.5	50.8	50.1	54.0	49.0	51.2	50.8
Age, median	34	39	50	51	49	44	50	46	49	54	35	50
IQR	29–42	32–51	39–65	39–66	36–61	34–58	37–63	36–55	37–63	40–68	31–42	37–62
Deprivation index	15.68	31.39	22.26	23.03	32.48	24.95	22.95	23.39	25.26	21.68	17.33	22.12
Diabetes, n	103	500	370	334	403	1,710	380	605	433	631	2	2,051
%	0.6	2.4	6.0	4.9	5.6	3.0	4.7	6.5	5.4	6.5	1.3	5.8
Alcohol misuse, n	70	1,083	86	614	587	2,440	219	107	565	175	8	1,074
%	0.4	5.3	1.4	9.0	8.2	4.2	2.7	1.1	7.0	1.8	5.3	3.0
Liver disease, n	34	128	40	99	58	359	154	75	41	79	1	350
(prevalent) %	1.0	1.6	0.8	1.9	1.1	1.3	2.5	1.2	0.7	1.0	0.8	1.3

### Referral and attendance

A total of 7183 patients were identified for further investigation (Table 2 and Fig 2): 715 were referred by a GP (Pathway 1), 4397 participants were identified from a risk group (Pathway 2), and 2071 responded to the AUDIT questionnaire (Pathway 3). In 2492/7183 notes were not examined, in the remainder, 2082/4701 (44%) were eligible and of these 910/2082 (44%) attended nurse assessment in total (Table 3 and Fig 2). There was a higher percentage of males (56.3%), reflecting the higher proportion of males in intervention practices at baseline, and the median age was 47 (IQR 31–58). Almost 75% of patients had a BMI corresponding to overweight or obese, and 74.5% had a waist grade of increased or substantially increased risk. According to the AUDIT questionnaire at nurse assessment, 42.6% of clinic attendees were drinking at hazardous, harmful or dependent levels (Table 3). The breakdown of participants per clinic and their diagnostic outcome is as follows:

**Table 2:** Route of invitation to study.

	Module 1 (GP referral)	Module 2 (Risk group)	Module 3 (AUDIT mailshot)	Total
Identified for further investigation (n, %)	715 (10.0)	4397 (61.2)	2072 (28.8)	7184 (100.0)
Invited to clinic (n, %)	627 (30.1)	1,235 (59.3)	220 (10.6)	2082 (100.0)
Attended clinic (n, %)	272 (29.9)	465 (51.1)	173 (19.0)	910 (100.0)

**Table 3:** Characteristics of patients who attended clinic.

Characteristic (N = 910)		
Gender Male (n, %)	512	56.3
Age (median, IQR)	47	31–58
BMI (median, IQR)	28.0	24.8–32.9
Weight (kg) (mean, SD)	84.0	19.9
Waist (cm) (mean, SD)	99.1	18.4
Waist grade (n, %)		
Normal	205	22.5
Increased risk	180	19.8
Substantially increased risk	498	54.7
Missing	27	3.0
AUDIT grade (n, %)		
Low Risk	513	56.4
Hazardous	236	25.9
Harmful	64	7.0
Dependent	88	9.7
Missing	9	1.0

#### Pathway 1

Out of the 715 potential participants referred by GPs and practice nurses, 627 (87.7%) were invited into the study, and a total of 272 (38.0%) took part (Fig 2 and Table 2). Half of this group had some evidence of liver disease (liver warning 25.8%, progressive fibrosis 19.2%, probable cirrhosis 5.2%, Table 4).

**Table 4:** Liver fibrosis by identification route.

	Module 1 (GP referral)	Module 2 (Risk group)	Module 3 (AUDIT mailshot)	Total
Liver stage, n (%)				
No fibrosis	135 (49.8)	248 (52.1)	122 (70.5)	505 (55.5)
Liver warning	70 (25.8)	116 (24.8)	34 (19.7)	220 (24.2)
Progressive fibrosis	52 (19.2)	76 (17.4)	13 (7.5)	141 (15.5)
Probable cirrhosis	14 (5.2)	26 (5.8)	4 (2.3)	44 (4.8)
Total	271 (100.0)	466 (100.0)	173 (100.0)	910 (100.0)

### Pathway 2

The majority of the randomly selected 4397 participants were identified due to abnormal blood tests (n = 2657), followed by diabetes (n = 942), CIRRUS algorithm (n = 665) and alcohol misuse (n = 163). 1235 (28%) participants were invited into the study with 465 attending. Nearly half showed some evidence of liver disease (47.9% in total–liver warning 24.8%, progressive fibrosis 17.4%, probable cirrhosis 5.8%).

#### Pathway 3

Of 9510 AUDIT questionnaires sent out, 2071 were returned altogether (21.8% response rate). After exclusions due to insufficient score (<8), 220 participants were invited to clinic with 173 (79%) included in the study. Most (70.5%) did not have evidence of liver fibrosis, with 19.7% having a liver warning, 7.5% progressive fibrosis and 2.3% probable cirrhosis.

Overall 910 cases had a validated liver diagnosis and were categorised: 44 (4.8%) probable cirrhosis, 141 (15.5%) progressive fibrosis, 220 (24.2%) liver warning and 505 (55.5%) with no evidence of liver fibrosis (Tables 4 and 5). A liver disease aetiology was ascribed in all but 12 (1.3%) cases, of whom one had probable cirrhosis and six progressive fibrosis. Almost half of the cases of liver disease identified in clinic were attributed to NAFLD (450, 49.5%), and a further 356 cases (39.1%) were due to alcohol. The remaining causes are displayed in Table 5. Out of the 405 with a liver disease diagnosis, 136 (33.6%) were from a GP referral, 218 (53.8%) from the risk factor pathway with only 51 (12.6%) from the AUDIT mailout.

**Table 5:** Liver stage by aetiology.

	Alcohol	Obesity / metabolic syndrome	Viral hepatitis	Autoimmune	Iron	Misc	Unknown aetiology	total
No fibrosis	242	249	1	1	6	0	6	505
Liver warning	89	102	4	4	2	1	18	220
Progressive fibrosis	44	75	3	5	4	4	6	141
Probable cirrhosis	16	24	1	0	2	0	1	44
Total	391	450	9	10	14	5	31	910

### Liver disease identification

Following the intervention period (July 2014-September 2016), a total of 221 cases of liver disease (0.8%) were identified by Read code search in the control arm compared to 287 cases of liver disease (1.0%) in the intervention arm for patients aged over 25 (Table 6). A further 257 cases of liver disease were identified in the nurse led clinic (not coded by practices) giving a total of 544 cases in the intervention arm compared to 221 in the control arm in the period July 2014-September 2016. A logistic regression of liver disease on randomised arm, adjusting for baseline liver disease rate gave an odds ratio of 2.4 (2.1, 2.8) in favour of the intervention arm. A sensitivity analysis, utilising just progressive fibrosis or probable cirrhosis in addition to Read codes as indicative of liver disease similarly gave an odds ratio of 1.8 (1.5, 2.1) in favour of the intervention arm (395 in intervention vs 221 in control).

**Table 6:** Number of new cases of liver disease identified by Read code and in clinic.

	Intervention			Control
How identified	In clinic	Not in clinic	Total	
Read coded	102 (0.37)	185 (0.67)	287 (1.03)	221 (0.83)
Not Read coded	257 (0.92)	—	257 (0.92)	—
Total	359 (1.29)	185 (0.67)	544 (1.95)	221 (0.83)

## Discussion

### Feasibility issues

This study aimed to assess the feasibility in primary care of different approaches in identifying subjects with progressive liver disease. We also designated a new category of subjects given a liver warning, subjects with modifiable behavioural risk factors for liver disease with borderline liver fibrosis results were advised to reduce their alcohol intake or lose weight. The strategies employed yielded a substantially higher total of new diagnoses in the intervention practices compared to the control– 544 versus 221. In the intervention practices each subject was given a stage of fibrosis and a disease aetiology confirmed in a virtual clinic, information not available for comparison in control practices. The study was also able to demonstrate that the incorporation of liver nurses into the practices was practical, straightforward to implement, and welcomed by the practice staff. This was reflected in that only three practices within the top 13 by deprivation index in Southampton declined to take part in the study, and eight out of the ten practices eventually involved were not previously research active. The good practice recruitment also reflected support for the programme as a whole including the GP referral pathway 1. The liver nurses were provided with system specific training within each practice which was quick to perform and a good rapport with the practice staff was established.

The strategies utilised to search for and recruit subjects varied in terms of their success rates on both counts. Most subjects that were referred from their GP (Pathway 1) were invited into the study, and although less than half ultimately attended clinic, this route of identification yielded the most new diagnoses and was less demanding logistically. Conversely, the AUDIT mailouts which required a greater degree of organisation produced the lowest response rate and the lowest proportion and yield of new diagnoses. Our findings suggest that this blanket sending of AUDIT questionnaires is not helpful as only one-fifth of recipients initially responded, and only 35% of these responders reported drinking at harmful or hazardous levels. Additionally, only 9.8% of this group were eventually found to have progressive fibrosis / probable cirrhosis, in comparison to 24.4% of those both referred by the practice healthcare team and identified in the risk groups. The majority of the newly identified cases of liver disease in this group were attributed to NAFLD, with slightly less due to alcohol. We found that response rates to the AUDIT questionnaires were lower in the most deprived practices which also conferred the highest prevalence of significant liver disease. These findings were in marked contrast to our previous study (ALDDeS) [19] where the response rate was 47%—the practices involved were based in more affluent suburban and rural settings with lower deprivation scores. In the LOCATE practices, selected on the basis that they had the highest levels of liver admissions, with less well educated patients with lower socio-economic status, postal screening was a less effective strategy.

Searching for at risk subjects (Pathway 2) produced a large number of records to review to ascertain suitability for invitation. If more than 300 patients per practice were identified as eligible, then a random sample of 300 was recruited. Although a large number of liver diagnoses were made in this group (47.9%), only 28% of the original number were invited into the study. This was due to the relatively complex nature of the process involving multiple screening of patient records. It was also noted that very few codes for alcohol misuse were identified (163/4397), suggesting that alcohol misuse, the major risk factor for progressive liver disease, is probably underreported and therefore more difficult to search for by code searches. As many of the subjects identified via this route are already likely to be being seen in other primary care chronic disease clinics, e.g. diabetes, it would seem plausible and relatively straightforward to include liver screening amongst the investigations carried out. We set out to recruit participants from this pathway who appeared to have the most severe abnormalities on pre-existing blood tests—this may have slightly increased the prevalence within this group and the study overall however this was also an important feasibility aim in determining most effective identification strategies.

As part of this study we set out to see if we could simplify the management of liver disease by reviewing all the collated study data centrally in a joint virtual clinic with a consultant hepatologist and a GP. Once the study was up and running we were assessing approximately 50 new subjects / month with two WTE nurses and less than half a consultant session. It was possible to ascribe a liver fibrosis stage in all subjects, and a disease aetiology in more than 98%. As a comparison in the combined UHS liver clinics of five consultant hepatologists and two specialist registrars, 1,038 new patients were assessed in 12 months (2016–7), 86 patients per month. The pick-up rate for progressive fibrosis and liver warning in the study group was almost identical to that found in patients seen in UHS liver clinics—a total of 1,915 clinic patients underwent elastography in UHS clinics between April 2012 and March 2016: 327 (17%) had cirrhosis, 328 (17%) progressive fibrosis, 470 (24%) liver warning and 767 (41%) were normal. The cirrhosis rate in the study was lower—4.8% v 17% but considering the UHS clinics include patients presenting symptomatically with liver disease this is perhaps not surprising. In our study utilising elastography was successful, with only a 1.9% failure rate in obtaining valid measurements. Our virtual clinic has advantages over conventional triage due to the fact that test data not typically generated by primary care to date (fibrosis markers and TE readings) were already available, in addition to a graphical representation of other blood test results over time on secondary care IT systems, thus allowing more time to be dedicated to subjects with more advanced disease. Within the constraints of a resource stretched healthcare service such as the NHS, this model rapidly allows the targeted emphasis onto subjects requiring of secondary care input without filling clinics with subjects who can safely be kept within primary care management.

### Comparison with existing literature

Significant CLD (liver warning, progressive fibrosis or cirrhosis) was seen in half of the participants referred by the practice healthcare team and half of the risk groups. The liver warning category was considered a borderline category at results in the lower amber range; subjects were informed that there was no definitive evidence of liver fibrosis, but for hazardous or harmful drinkers, it would be an indication to reduce alcohol intake within the recommended guideline of 14 units / week, and for obese patients to lose weight. When compared to the overall background prevalence of identified CLD within our practice population at 1.3% (aged ≥25), our findings suggest a significant number of undetected cases exist within the community, particularly among at risk groups. This finding has been echoed in previous studies looking at persons with both NAFLD [28] and hazardous alcohol use [22].

Appropriate referral of patients to secondary care is key to minimise cost and resource use. This study used two different investigative modalities, TE and serum fibrosis markers. There is evidence that improved diagnostic accuracy can be achieved by using both [25] and this approach has been suggested for use in primary care [29]. Furthermore, a recent large study assessing non-invasive markers of fibrosis in NAFLD found that TE had the highest AUROC for advanced fibrosis (F ≥ 3), along with a fibrosis blood panel FibroMeter^V2G^ which includes hyaluronic acid as one of the parameters, compared with blood test panels consisting of other direct fibrosis markers and/or indirect markers [30]. This is especially relevant to our study as the majority of liver disease identified was thought to be due to NAFLD. NICE guidelines published in 2016 [30] suggest the use of the Enhanced Liver Fibrosis (ELF) Test in NAFLD, a test which comprises both HA and P3NP.

A community study looking at patients with type 2 diabetes to assess for advanced fibrosis using TE in 705 subjects found significant fibrosis (≥8 kPa) in 12.7% and cirrhosis (≥13 kPa) in 2.1% [31]. These findings are similar when compared to our data looking exclusively at TE outcomes–in our study, 110/910 (12.1%) participants had a measurement of ≥8 kPa representing progressive fibrosis, however 36 (4.0%) were above the 13kPa cut off for cirrhosis. This difference may be due to the fact that we were more inclusive and not restricted only to those with diabetes. A further study assessing diabetic patients with NAFLD for significant liver fibrosis found 17.1% affected, albeit from hospital diabetes outpatient clinics which probably represents a more severe disease spectrum [32]. Nevertheless, it is clear that the prevalence of significant fibrosis in communities with liver risk factors is substantial and could be incorporated into the practitioners’ approach to both chronic disease management and alcohol misuse. Success rates using elastography were similar to the studies [31, 32] discussed above.

### Strengths and limitations

This was a large feasibility study carried out over an urban primary care population in the United Kingdom, with the population of the practices involved conferring the highest rates of liver admissions to the local General Hospital. The modalities used were unique in the sense that this is the first study to have been carried out using both a non-invasive fibrosis marker panel (STL) and transient elastography together, culminating in nearly half of the participants having a degree of CLD including 20.3% with progressive fibrosis or cirrhosis. We have introduced ‘liver warning’ as a way of dealing with those with borderline results/evidence of early fibrosis, this enables advice regarding lifestyle modification to prevent progression, and/or as a prompt to look for alternative causes e.g. viral hepatitis. It remains to be seen if a sustained reduction of risk and positive influence on health outcomes in the longer term occurs.

A further strength of this study is the demonstration of the ease of carrying out clinics within the practices. Almost all participants were seen at their usual GP practice, with valid and reliable elastography measurements, similar to other published work, possible using a portable scanning device. This approach allowed the majority of participants to be managed within the community with appropriate referral if required.

Our study has several limitations. There were some differences within the baseline characteristics of the intervention and control practices. This is likely due to an increased student population in one of the intervention practices. However, the prevalence of pre-existing liver disease between intervention and control groups was the same. We also did not subject any of the participants to a liver biopsy due to the pragmatic design of the study and so we do not have any histological diagnoses to back up our investigative results. Nevertheless, every case which suggested liver warning or more advanced disease was reviewed by a hepatologist, with further investigation arranged if required. Additionally, as this was a study focused on enhancement of identification of disease, i.e. diagnosis, we have not been in a position to follow up participants longitudinally and are therefore unable to comment on if such early diagnoses translate into better outcomes. Logically this would appear to be the case: brief interventions in primary care for alcohol have been shown to reduce drinking levels [33], and coupled with feedback on liver health/diagnosis one may see an even greater improvement.

As this was a feasibility study a full formal health economic assessment was not performed, but the service costing for the UHS business case was around one third that of the existing liver outpatient referral service: the virtual clinic approach was costed by the commissioning team at University Hospitals as part of a business case for an NHS based service with a proposed tariff of £108 in comparison with £361 for a secondary care liver referral with two outpatient attendances including liver elastography.

### Implications for practice

This is a large feasibility study, demonstrating the benefit of utilising available technologies within a primary care setting to increase the early detection of liver disease. At present, screening liver disease is not part of routine practice within UK primary care, and given pressure of work and prioritisation of incentivised activities it is either overlooked or not recognised. There would be opportunity to assess liver health, for example during routine diabetic reviews and NHS health checks which are offered from those aged 40–74 and assess BMI, alcohol intake as well as risk of diabetes. The upskilling of front line staff, e.g. specialist nurses in delivering these health checks to incorporate liver health training may be considered a reasonable option as it would allow both continuity of care and delivering of related health strategies. A larger trial of our approach (without blanket AUDIT mailouts), with longer term follow up including liver outcomes as well as health economic analysis is planned. Alternative strategies to identify and screen harmful drinkers to assess those at higher risk remains a priority.

## Supporting information

S1 Appendix Audit queries in full. (DOCX)
