# Supplementary material for: Local care and treatment of liver disease (LOCATE) – A cluster-randomized feasibility study to discover, assess and manage early liver disease in primary care
Source: PLoS One. 2018 Dec 21;13(12):e0208798. doi: 10.1371/journal.pone.0208798 (PMC6303066; doi:10.1371/journal.pone.0208798)
Supplement: S1 Appendix — (DOCX) [file pone.0208798.s001.docx]

**A description of the 26 .HQL files written by Explore Health**

**for the initial baseline audit of the 10 GP Practices in LOCATE study**

**Colin Newell, 17jun2014**

Original queries are written in HQL (Health Query Language, part of MIQUEST) by Burrinder Grewal of Explore Health ([burrinder@explore-health.com](mailto:burrinder@explore-health.com)).

The queries are in two versions, one using Read V2 codes and one using Read V3. Both codes are documented in this description. The queries are sorted, in this document, into the same order as they are listed in both **SETINFO.INI** and per the “*QRY_ORDER, 0nn” line in the header of each query. i.e. they are executed in the order they appear in the document.

**Standard header**

Every query begins with a 10 line near-identical header section like this.

*QRY_WDATE, 20140117, 17/01/2014

*QRY_SDATE, 20140117, 17/01/2014

*QRY_TITLE, LFTINC, LFT Inclusion

*QRY_ORDER, 004

*QRY_AGREE, LOCAL,

*QRY_SETID, LIVERV3, LIVER V3

*ENQ_IDENT, LOCAL, Local enquirer

*QRY_MEDIA, D, DISK

*ENQ_RSPID, LOCAL,

*QRY_CODES, 0, 9999R2, Read version 2 (*or R3 for the read 3 versions of the queries)*

Only the title and order vary. Nothing else. Hence in this document I have removed the header from each

**Summary of steps**

1. Merge POPM and POPF to produce POPALL All patients ever Registered at the practice, ages 0-125
2. Generate from POPALL report PATALL (Columns: Practice, NHS No., Age, DoB, Sex)
3. Merge LFTINC, ALCINC, LVRDIAG to produce STDYGRP Patients with alcohol related problem jul2012-jul2013
4. Generate from STDYGRP reports PATDEMO, COMORB, BLOOD1-13 and ALCCON

**Overview of output reports**

All output reports/datasets generated by these queries except PATALL refer to “The Study Group”. This is defined as patients who have recorded one or more Read codes relating to LFT, ALC, or Liver disease diagnosis during the year jul2012 thru jun2013. The precise V2 and V3 Read codes used to identify the Study Group are listed in the three queries LFTINC, ALCINC, and LVRDIAG below.

1. **PATALL**

Rows: One row per patient. All patients, all ages, ever registered at the Practice

Cols: *Practice, NHS No, Age, DoB, Sex*

1. **PATDEMO**

Rows: “Study group” patient ‘Demographics’

Info covering “PAL Care QOF V23, Type2 Diabetes, BMI, HMG COA reductase inhibitor,

Alcohol, pre-existing liver abnormalities…

Possibly each section is in a separate dataset??)

Cols: *Practice, NHS_No., Age (as at 31dec2013) DoB, Sex*

1. **COMORB**

Rows: Comorbidities recorded jul2012 – jun2013 for “Study Group” patients

Cols: *NHS_No., ReadCode, Rubric, Date*

1. **BLOOD** (13 files, each covering 6m i.e. total 6.5 years**)**

Rows: Blood test results recorded 1jul2007 thru 31dec2013 for “Study Group” patients

Cols: *NHS_No., ReadCode, Rubric, Date, Value1, Value2*

1. **ALCCON**

Rows: Alcohol consumption recorded jul2012 – jun2013 for “Study Group” patients  (Read V3 code 136..00)

Cols: *NHS_No., ReadCode, Rubric, Date, Value1, Value2*

**1. POPM - Male Population**

Query is identical for Read V2 and Read V3

DEFINE AGE AS @YEARS(‘31/12/2013’, DATE_OF_BIRTH)

SUBSET POPM TEMP

FROM PATIENTS

WHERE AGE IN (‘0’ - ‘125’)

AND SEX IN (‘M’)

**2. POPF - Female Population**

Query is identical for Read V2 and Read V3

DEFINE AGE AS @YEARS(‘31/12/2013’, DATE_OF_BIRTH)

SUBSET POPF TEMP

FROM PATIENTS

WHERE AGE IN (‘0’ - ‘125’)

AND SEX IN (‘F’)

**3. POPALL - All Population**

Query is identical for Read V2 and Read V3

FOR POPM OR POPF

SUBSET POPALL TEMP

FROM PATIENTS

**4. LFTINC - LFT Inclusion**

**a) Read V2**

FOR POPALL

SUBSET LFTINC TEMP

FROM JOURNALS (ONE FOR PATIENT)

WHERE CODE IN (‘1B1K’, ‘339W’, ‘42R5’, ‘43W0’,

‘442L’, ‘44CO’, ‘44E3’, ‘44E4’,

‘44E40’, ‘44E7’, ‘44E9’, ‘44EA’,

‘44EB’, ‘44F’, ‘44g3’, ‘44l’,

‘44M4’, ‘467C’, ‘4J44’, ‘4KK1’,

‘4Q’, ‘4Q3’, ‘4QC’, ‘4QCE0’,

‘79250’, ‘7A238’, ‘7G2AD’, ‘9EMZ’,

‘9Oe2’, ‘A7073’, ‘A70z1’, ‘C253’,

‘C371’, ‘C3762’, ‘G858’, ‘J612’,

‘J613’, ‘J615z’, ‘J61y1’, ‘J61y4’,

‘J61y7’, ‘J61y8’, ‘J64’, ‘J651z’,

‘J6711’, ‘K59’, ‘PD35’, ‘SP0’,

‘19E7’, ‘222’, ‘2F5’, ‘448T’,

‘44E8’, ‘44EC’, ‘44ED’, ‘44g’,

‘44gA’, ‘44GB’, ‘44lL’, ‘44MI’,

‘4618’, ‘46P2’, ‘4Q9’, ‘4QCE’,

‘4QCE1’, ‘72182’, ‘782M’, ‘7A6My’,

‘7H605’, ‘8IAZ’, ‘9E3’, ‘9NS’,

‘A703’, ‘A707’, ‘A70z0’, ‘A97’,

‘B577’, ‘C3500’, ‘C3714’, ‘E0112’,

‘F430’, ‘J61’, ‘J614’, ‘J6160’,

‘J61z’, ‘J6330’, ‘J6510’, ‘J661’,

‘J6617’, ‘J6705’, ‘K3110’, ‘L22y’,

‘PB6’, ‘PF318’, ‘SKz’,

‘44E%’, ‘44F3%’)

AND DATE IN (‘01/07/2012’ - ‘30/06/2013’)

**b) Read V3**

FOR POPALL

SUBSET LFTINC TEMP

FROM JOURNALS (ONE FOR PATIENT)

WHERE CODE IN ( ‘44M4.’, ‘44MI.’, ‘X772i’, ‘XaIRc’, ‘XE2eA’,

‘44F3.%’, ‘XE28S’, ‘44F..’,

‘44G..’, ‘44G3.’, ‘44GA.’, ‘44GB.’,

‘X771e’, ‘X771f’, ‘XaIRi’, ‘XaLJx’, ‘XE25P’, ‘X771d’,

‘X80DL’, ‘44E..%’, ‘4QCE.’, ‘4QCE0’, ‘4QCE1’, ‘Xa972’, ‘XaERu’,

‘XaERv’, ‘XaERw’, ‘XaES7’, ‘XaESA’, ‘XaETf’, ‘XaX4F’,

‘XaX4W’, ‘XaX4X’, ‘XE2mf’, ‘XE2qu’, ‘XE28O’,

‘A70z0’, ‘B577.’, ‘C3500’, ‘C371.’, ‘C3714’, ‘C3762’,

‘E0112’, ‘J61..’, ‘J613.’, ‘J614.’, ‘J6160’, ‘J61y1’,

‘J61y7’, ‘J61z.’, ‘J6510’, ‘J651z’, ‘J661.’, ‘J6617’,

‘X0053’, ‘X306i’, ‘X306S’, ‘X3071’, ‘X307L’, ‘X307M’,

‘X307W’, ‘X3089’, ‘X308A’, ‘X308i’, ‘X308u’, ‘XaE6u’,

‘XaQIT’, ‘XE0b4’, ‘XE0eN’, ‘XE0RK’)

AND DATE IN (‘01/07/2012’ - ’30/06/2013’)

**5. ALCINC - ALC Inclusion**

**a) Read V2**

FOR POPALL

SUBSET ALCINC TEMP

FROM JOURNALS (ONE FOR PATIENT)

WHERE CODE IN (‘136W’, ‘1B64’, ‘72182’, ‘7447’, ‘74510’, ‘7J02C’,

‘302’, ‘BB02’, ‘C253’, ‘C3714’, ‘E0112’, ‘E012’,

‘E01y0’, ‘E03y2’, ‘E230’, ‘E231’, ‘E23z’, ‘E250’,

‘E2A0’, ‘E2D2’, ‘Eu06y’, ‘Eu104’, ‘Eu108’, ‘F2510’,

‘F25B’, ‘J153’, ‘J612’, ‘J613’, ‘J63’, ‘J6710’,

‘S737’)

AND DATE IN (‘01/07/2012’ - ‘30/06/2013’)

**b) Read V3**

FOR POPALL

SUBSET ALCINC TEMP

FROM JOURNALS (ONE FOR PATIENT)

WHERE CODE IN ( ‘C3714’, ‘E0112’, ‘E01y0’, ‘E230.’, ‘E23z.’, ‘Eu104’,

‘J153.’, ‘J613.’, ‘J63..’, ‘X0053’, ‘X006u’, ‘X00Rm’,

‘X3071’, ‘X308v’, ‘Xa1yZ’, ‘XaLWu’, ‘XE0b4’, ‘XE1Xu’,

‘XE1YQ’, ‘XE1YX’)

AND DATE IN (‘01/07/2012’ - ’30/06/2013’)

**6. LFTALC - LFT ALC Group**

Query is identical for Read V2 and Read V3

FOR LFTINC OR ALCINC

SUBSET LFTALC TEMP

FROM JOURNALS (ONE FOR PATIENT)

**7. LVRDIAG - Liver Disease Diagnosis in last 18 months (sic, but note date range is actually 12m)**

**a) Read V2**

FOR POPALL

SUBSET LVRDIAG TEMP

FROM JOURNALS (ONE FOR PATIENT)

WHERE CODE IN (‘J6%’, ‘PB6%’)

AND DATE IN (‘01/07/2012’ - ’30/06/2013’)

**b) Read V3**

FOR POPALL

SUBSET LVRDIAG TEMP

FROM JOURNALS (ONE FOR PATIENT)

WHERE CODE IN (‘X306R%’)

AND DATE IN (‘01/07/2012’ - ’30/06/2013’)

**8. STDYGRP - Study Group**

Query is identical for Read V2 and Read V3

FOR LFTALC OR LVRDIAG

SUBSET STDYGRP TEMP

FROM JOURNALS (ONE FOR PATIENT)

**9. PATDEMO - Patient Demography Report**

**a) Read V2**

DEFINE AGE AS @YEARS(‘31/12/2013’, DATE_OF_BIRTH)

FOR STDYGRP

REPORT FIXED

PRINT PRACTICE, NHS_NUMBER, AGE, DATE_OF_BIRTH, SEX

FROM PATIENTS

#PAL Care QOF V23

PRINT CODE, RUBRIC, DATE

FROM JOURNALS (LATEST FOR PATIENT)

WHERE CODE IN (‘1Z01’, ‘2JE’, ‘8BA2’, ‘8BAP’, ‘8BAS’, ‘8BAT’,

‘8BAe’, ‘8BJ1’, ‘8CM1%’, ‘8CM4’, ‘8CME’, ‘8H6A’,

‘8H7L’, ‘8H7g’, ‘8HH7’, ‘8IEE’, ‘9EB5’, ‘9Ng7’,

‘ZV57C’, ‘8CMQ’, ‘9NgD’)

#Type II diabetes

PRINT CODE, DATE

FROM JOURNALS (EARLIEST FOR PATIENT)

WHERE CODE IN (‘C10F%’, ‘C109J’, ‘C10FJ’)

#BMI

PRINT CODE, DATE, VALUE1

FROM JOURNALS (LATEST FOR PATIENT)

WHERE CODE IN (‘22K’)

#HMG COA reductase inhibitor

PRINT CODE, DATE, VALUE1, VALUE2

FROM JOURNALS (EARLIEST FOR PATIENT)

WHERE CODE IN (‘bx%’, ‘8I27’, ‘8I3C’,

‘8I63’, ‘8I76’, ‘TJC24’, ‘TJC25’, ‘U60CA’)

#Alcohol - No intake data

PRINT CODE, RUBRIC, DATE, VALUE1

FROM JOURNALS (EARLIEST FOR PATIENT)

WHERE CODE IN (‘C3714’, ‘E0112’, ‘E01y0’, ‘E230’, ‘E23z’, ‘Eu104’,

‘J153’, ‘J613’, ‘J63’,

‘136S’, ‘136T’, ‘136K’)

AND DATE IN (‘01/07/2007’ - ‘30/06/2012’)

#Referral to secondary care services

# too wide, also can only have 1 record per row

#Hospital admissions with liver disease

PRINT CODE, RUBRIC, DATE

FROM JOURNALS (EARLIEST FOR PATIENT)

WHERE CODE IN (‘8Hk5’, ‘8HkM’, ‘9NlN’, ‘7P0D%’, ‘8H2H’)

#Pre-existing abnormal liver test

PRINT CODE, RUBRIC, DATE

FROM JOURNALS (EARLIEST FOR PATIENT)

WHERE CODE IN (‘44D2’)

#Incident Liver abnormalities

PRINT CODE, RUBRIC, DATE

FROM JOURNALS (LATEST FOR PATIENT)

WHERE CODE IN (‘25G3’, ‘25G4’, ‘25H4’, ‘25H5’, ‘25H6’,

‘14C5’, ‘25H9’)

**b) Read V3**

DEFINE AGE AS @YEARS(‘31/12/2013’, DATE_OF_BIRTH)

FOR STDYGRP

REPORT FIXED

PRINT PRACTICE, NHS_NUMBER, AGE, DATE_OF_BIRTH, SEX

FROM PATIENTS

#PAL Care QOF V23

PRINT CODE, RUBRIC, DATE

FROM JOURNALS (LATEST FOR PATIENT)

WHERE CODE IN (‘1Z01.’, ‘XaQg1’, ‘8BA2.%’, ‘XaIse%’,

‘XaIpI’, ‘XaMhi’, ‘XaJv2%’, ‘8H6A.’, ‘8H7L.’, ‘XaAex’, ‘XaIlk’,

‘XaAg6’, ‘XaAT5’, ‘XaEJE%’, ‘XaAWN’, ‘XaAPW’, ‘XaRFG’, ‘XaRFF’,

‘9EB5.’, ‘ZV57C’, ‘XaXUG’, ‘XaXoP’, ‘XaXoW’, ‘XaYRB’, ‘XaYRD’,

‘XaYRy’, ‘XaYpV’)

#Type II diabetes

PRINT CODE, DATE

FROM JOURNALS (EARLIEST FOR PATIENT)

WHERE CODE IN (‘C10F.%’, ‘X40J5%’, ‘X40J6’)

#BMI

PRINT CODE, DATE, VALUE1

FROM JOURNALS (LATEST FOR PATIENT)

WHERE CODE IN (‘22K..’)

#HMG COA reductase inhibitor

PRINT CODE, DATE, VALUE1, VALUE2

FROM JOURNALS (EARLIEST FOR PATIENT)

WHERE CODE IN (‘x01R1%’, ‘8I27.’, ‘8I3C.’,

‘8I63.’, ‘8I76.’, ‘TJC24’, ‘TJC25’, ‘U60CA’)

#Alcohol - No intake data

PRINT CODE, RUBRIC, DATE, VALUE1

FROM JOURNALS (EARLIEST FOR PATIENT)

WHERE CODE IN (‘C3714’, ‘E0112’, ‘E01y0’, ‘E230.’, ‘E23z.’, ‘Eu104’,

‘J153.’, ‘J613.’, ‘J63..’, ‘X0053’, ‘X006u’, ‘X00Rm’,

‘X3071’, ‘X308v’, ‘Xa1yZ’, ‘XaLWu’, ‘XE0b4’, ‘XE1Xu’,

‘XE1YQ’, ‘XE1YX’, ‘136S.’, ‘136T.’, ‘136K.’,

‘XaKvB’, ‘XaKvA’, ‘XaXje’, ‘XaXjd’)

AND DATE IN (‘01/07/2007’ - ’30/06/2013’)

#Referral to secondary care services

# too wide, also can only have 1 record per row

#Hospital admissions with liver disease

PRINT CODE, RUBRIC, DATE

FROM JOURNALS (EARLIEST FOR PATIENT)

WHERE CODE IN (‘8Hk5.’, ‘8HkM.’, ‘9NlN.’, ‘7P0D%’,

‘XaLrh’, ‘XaLav%’, ‘X70jl%’,

‘XaATd’, ‘XaAgd’, ‘XaAMP’, ‘XaAQG’, ‘XaAak’, ‘8H2H.’)

#Pre-existing abnormal liver test

PRINT CODE, RUBRIC, DATE

FROM JOURNALS (EARLIEST FOR PATIENT)

WHERE CODE IN (‘44D2.’)

#Incident Liver abnormalities

PRINT CODE, RUBRIC, DATE

FROM JOURNALS (LATEST FOR PATIENT)

WHERE CODE IN (‘25G3.’, ‘25G4.’, ‘25H4.’, ‘25H5.’, ‘25H6.’,

‘14C5.’, ‘25H9.’, ‘Xa7UJ’, ‘Xa7UP’, ‘Xa7UQ’)

**10. COMORB - Patient Comorbidity Report**

**a) Read V2**

DEFINE AGE AS @YEARS(‘31/12/2013’, DATE_OF_BIRTH)

FOR STDYGRP

REPORT

PRINT NHS_NUMBER, CODE, RUBRIC, DATE

FROM JOURNALS (ALL FOR PATIENT)

WHERE CODE IN (‘136W’, ‘1451’, ‘1B1K’,

‘1B1L’, ‘1B64’,

‘2F5%’, ‘46M4’, ‘72182’, ‘725’,

‘731’, ‘7313’, ‘74’, ‘74431’,

‘7447’, ‘745’, ‘74510’, ‘74530’,

‘782M’, ‘79250’, ‘7J02C’, ‘8IAZ’,

‘A703’, ‘A707’, ‘A7073’, ‘A70z0’,

‘A70z1’, ‘A97’, ‘B302’,

‘B577’, ‘BB02’, ‘BBE0’,

‘C10’, ‘C108y’, ‘C1097’, ‘C109C’,

‘C10F’, ‘C253’, ‘C3500’,

‘C371’, ‘C3714’, ‘C3762’, ‘C380’,

‘D00’, ‘D3121’, ‘D315’,

‘E0112’, ‘E012’, ‘E01y0’, ‘E030’,

‘E03y2’, ‘E1137’, ‘E2002’, ‘E2003’,

‘E200z’, ‘E204’, ‘E21z’, ‘E230’,

‘E231’, ‘E23z’, ‘E241’, ‘E250’,

‘E264’, ‘E28’, ‘E2A0’, ‘E2D2’,

‘Eu06y’, ‘Eu104’, ‘Eu108’, ‘F2510’,

‘F25B’, ‘F430’, ‘G410’, ‘G573’,

‘G858’, ‘J’, ‘J03’, ‘J153’,

‘J40’, ‘J61’, ‘J612’, ‘J613’,

‘J614’, ‘J615z’, ‘J6160’, ‘J61y1’,

‘J61y4’, ‘J61y7’, ‘J61y8’, ‘J61z’,

‘J63’, ‘J6330’, ‘J64’, ‘J6510’,

‘J651z’, ‘J661’, ‘J6617’, ‘J670’,

‘J6705’, ‘J670z’, ‘J6710’, ‘J6711’,

‘K3110’, ‘K59’, ‘L13’, ‘M161z’,

‘M2z0’, ‘PB6%’, ‘S737’)

AND DATE IN (‘01/07/2007’ - ’30/06/2013’)

**b) Read V3**

DEFINE AGE AS @YEARS(‘31/12/2013’, DATE_OF_BIRTH)

FOR STDYGRP

REPORT

PRINT NHS_NUMBER, CODE, RUBRIC, DATE

FROM JOURNALS (ALL FOR PATIENT)

WHERE CODE IN (‘1B1L.’, ‘A70z0’, ‘B577.’, ‘C10..’,

‘C108y’, ‘C1097’, ‘C3500’, ‘C371.’,

‘C3714’, ‘C3762’, ‘C380.’, ‘D315.’,

‘E0112’, ‘E01y0’, ‘E1137’, ‘E2002’,

‘E200z’, ‘E230.’, ‘E23z.’, ‘Eu104’,

‘G410.’, ‘G573.’, ‘J153.’, ‘J61..’,

‘J613.’, ‘J614.’, ‘J6160’, ‘J61y1’,

‘J61y7’, ‘J61z.’, ‘J63..’, ‘J6510’,

‘J651z’, ‘J661.’, ‘J6617’, ‘J670.’,

‘L13..’, ‘M161z’, ‘X0053’, ‘X006u’,

‘X00Rm’, ‘X00Rv’, ‘X00Sb’, ‘X306i’,

‘X306S’, ‘X3071’, ‘X307L’, ‘X307M’,

‘X307W’, ‘X3089’, ‘X308A’, ‘X308i’,

‘X308u’, ‘X308v’, ‘X40J5’, ‘X75uf’,

‘Xa1yZ’, ‘XaE6u’, ‘XaF05’, ‘XaLWu’,

‘XaQIT’, ‘XE0b4’, ‘XE0bH’, ‘XE0eN’,

‘XE0RK’, ‘XE13c’, ‘XE1Xu’, ‘XE1YC’,

‘XE1Ym’, ‘XE1YQ’, ‘XE1YX’, ‘XE2QL’)

AND DATE IN (‘01/07/2007’ - ’30/06/2013’)

**11. – 23. BLOOD1 – BLOOD13 - Patient Blood Test Results Report 1 thru 13**

**All 13 files are identical except for QRY_ORDER 11 thru 23 ‘AND DATE IN … ‘ last line.**

**Total range covered is 1 Jul 2007 to 31 Dec 2013. i.e. 6.5 years. Each file covers a 6 month period Jan-Jun or Jul-Dec.**

**a) Read V2**

DEFINE AGE AS @YEARS(‘31/12/2013’,DATE_OF_BIRTH)

FOR STDYGRP

REPORT

PRINT NHS_NUMBER, CODE, RUBRIC, DATE, VALUE1, VALUE2

FROM JOURNALS (ALL FOR PATIENT)

WHERE CODE IN (‘44G’, ‘44G3’, ‘44GA’, ‘44GB’,

‘44F3%’, ‘44F’, ‘44H5’, ‘44H50’, ‘44H51’, ‘44H52’, ‘44HB’, ‘44HC’,

‘44G7’, ‘44G9’, ‘44G4’, ‘42P’, ‘42P7’, ‘44M4’, ‘44MI’, ‘44E%’,

‘4QCE’, ‘4QCE0’, ‘4QCE1’, ‘44h1’, ‘44h6’, ‘44I5’,

‘44I50’, ‘44I51’, ‘44W4’, ‘43an’,

‘44h0’, ‘44h8’, ‘44I4’, ‘44I40’, ‘44I41’, ‘44I42’,

‘44J’, ‘44J1’, ‘44J2’, ‘44J8’, ‘44J9’, ‘44JA’, ‘44JZ’,

‘44J3%’, ‘44JC’, ‘44JD’, ‘44JF’,

‘423%’, ‘42H’, ‘42H1’, ‘42H5’, ‘42HZ’, ‘42I’, ‘42I1’, ‘42IZ’,

‘44M3’, ‘44M30’, ‘44M31’, ‘44MA’, ‘42A’, ‘42W%’,

‘42c%’, ‘44TB’, ‘44TC’, ‘44TL’,

‘42QE0’, ‘42QE1’, ‘42QE’, ‘43av’, ‘43m3’, ‘43mE’, ‘43p’, ‘43G1’,

‘43r4’, ‘43G2’, ‘43G3’, ‘43r7’, ‘42d4’, ‘42R4’, ‘42R9’,

‘42RA’, ‘42H%’, ‘43GP’, ‘43G4’, ‘43a4’, ‘42R7’, ‘42R5’,

‘42d1’, ‘42R6’)

AND DATE IN (‘01/07/2013’ - ‘31/12/2013’)

**b) Read V3**

DEFINE AGE AS @YEARS(‘31/12/2013’, DATE_OF_BIRTH)

FOR STDYGRP

REPORT

PRINT NHS_NUMBER, CODE, RUBRIC, DATE, VALUE1, VALUE2

FROM JOURNALS (ALL FOR PATIENT)

WHERE CODE IN (‘44G..’, ‘44G3.’, ‘44GA.’, ‘44GB.’, ‘X771e’, ‘X771f’, ‘XaIRi’,

‘XaLJx’, ‘XE25P’, ‘X771d’, ‘X80DL’, ‘44F3.%’, ‘XE28S’, ‘44F..’,

‘44H5.’, ‘44H50’, ‘44H51’, ‘44H52’, ‘44HB.’, ‘44HC.’, ‘X771i’, ‘XaES6’, ‘XE25Q’,

‘X80De’, ‘44G7.’, ‘44G9.’, ‘44G4.’, ‘XaES3’, ‘XaES4’, ‘X80E1’, ‘XE28U’, ‘42P..’,

‘42P7.’, ‘XaIwC’, ‘44M4.’, ‘44MI.’, ‘X772i’, ‘XaIRc’, ‘XE2eA’, ‘44E..%’, ‘4QCE.’,

‘4QCE0’, ‘4QCE1’, ‘Xa972’, ‘XaERu’, ‘XaERv’, ‘XaERw’, ‘XaES7’, ‘XaESA’, ‘XaETf’,

‘XaX4F’, ‘XaX4W’, ‘XaX4X’, ‘XE2mf’, ‘XE2qu’, ‘XE28O’, ‘44h1.’, ‘44h6.’, ‘44I5.’,

‘44I50’, ‘44I51’, ‘44W4.’, ‘XaIRf’, ‘XE2q0’, ‘XaDva’, ‘X7716’, ‘XE25d’, ‘43an.’,

‘44h0.’, ‘44h8.’, ‘44I4.’, ‘44I40’, ‘44I41’, ‘44I42’, ‘XE2pz’, ‘XaIRl’, ‘XaDvZ’,

‘44J..’, ‘44J1.’, ‘44J2.’, ‘44J8.’, ‘44J9.’, ‘44JA.’, ‘44JZ.’, ‘X771P’, ‘XM0lt’,

‘XaDvl’, ‘44J3.%’, ‘44JC.’, ‘44JD.’, ‘44JF.’, ‘XaERX’, ‘XE2q5’, ‘XaETQ’, ‘XaERc’,

‘423..%’, ‘Xa96v%’, ‘42H..’, ‘42H1.’, ‘42H5.’, ‘42HZ.’, ‘42I..’, ‘42I1.’, ‘42IZ.’,

‘44M3.’, ‘44M30’, ‘44M31’, ‘44MA.’, ‘Xa9wj’, ‘XE2e9’, ‘XE2eC’, ‘42A..’, ‘42W..%’,

‘42c..%’, ‘44TB.’, ‘44TC.’, ‘44TL.’, ‘X80U3%’, ‘X772q%’, ‘XaBLm%’, ‘XE24t%’, ‘XaCES%’,

‘XaJNg’, ‘42QE0’, ‘42QE1’, ‘42QE.’, ‘43av.’, ‘43m3.’, ‘43mE.’, ‘43p..’, ‘43G1’,

‘XaLV8’, ‘XaIZI’, ‘Xa7Yr’, ‘XaIZJ’, ‘43r4.’, ‘43G2.’, ‘XE2pg’, ‘XaJqq’, ‘43G3.’,

‘43r7.’, ‘XE2ph’, ‘XaJqp’, ‘42d4.’, ‘42R4.’, ‘XaItW’, ‘XE24r’, ‘X76t9’, ‘42R9.’,

‘42RA.’, ‘XaJm0’, ‘XaFvN’, ‘XaJph%’, ‘XaJoT’, ‘XaJpg%’,

‘X80bR’, ‘Xa0uu%’, ‘Xa9L4%’, ‘Xa9pg%’, ‘XaEKW%’, ‘XaFsF%’, ‘XaIvA%’,

‘XaPIL’, ‘XaPOp’, ‘XE259%’, ‘XE25A’, ‘XaIdY’, ‘XaIdZ’, ‘42H..%’,

‘43GP.’, ‘43G4.’, ‘43a4.’, ‘42R7.’, ‘42R5.’, ‘42d1’,

‘XaEPp’, ‘XE27a’, ‘42R6.’, ‘X76tH’, ‘XaERN’)

AND DATE IN (‘01/07/2013’ - ’31/12/2013’)

**24. ALCCON - Patient Alcohol Consumption Report**

**a) Read V2**

DEFINE AGE AS @YEARS(‘31/12/2013’, DATE_OF_BIRTH)

FOR STDYGRP

REPORT

PRINT NHS_NUMBER, CODE, RUBRIC, DATE, VALUE1, VALUE2

FROM JOURNALS (ALL FOR PATIENT)

WHERE CODE IN (‘136%’)

AND DATE IN (‘01/07/2012’ - ’30/06/2013’)

**b) Read V3**

DEFINE AGE AS @YEARS(‘31/12/2013’, DATE_OF_BIRTH)

FOR STDYGRP

REPORT

PRINT NHS_NUMBER, CODE, RUBRIC, DATE, VALUE1, VALUE2

FROM JOURNALS (ALL FOR PATIENT)

WHERE CODE IN (‘136..%’)

AND DATE IN (‘01/07/2012’ - ’30/06/2013’)

**25. PATALL - Pat Demo Report (cn: should read ‘All Patients Report’)**

Query is identical for Read V2 and Read V3

DEFINE AGE AS @YEARS(‘31/12/2013’, DATE_OF_BIRTH)

FOR POPALL

REPORT FIXED

PRINT PRACTICE, NHS_NUMBER, AGE, DATE_OF_BIRTH, SEX

FROM PATIENTS
